# The Influence of Pre-Exercise Glucose versus Fructose Ingestion on Subsequent Postprandial Lipemia

**DOI:** 10.3390/nu10020149

**Published:** 2018-01-29

**Authors:** Tsung-Jen Yang, Chih-Hui Chiu, Mei-Hui Tseng, Cheng-Kang Chang, Ching-Lin Wu

**Affiliations:** 1 Department of Physical Education, National Taiwan Normal University, Taipei 106, Taiwan; andy32437@yahoo.com.tw; 2 Graduate Program in Department of Exercise Health Science, National Taiwan University of Sport, Taichung 404, Taiwan; loveshalom@hotmail.com; 3 Sport Science Research Center, National Taiwan University of Sport, Taichung 404, Taiwan; tsengmh2009@gmail.com (M.-H.T.); wspahn@seed.net.tw (C.-K.C.); 4 Graduate Institute of Sports and Health Management, National Chung Hsing University, Taichung 402, Taiwan; psclw@dragon.nchu.edu.tw

**Keywords:** glycemic index, triacylglycerol, high-density lipoprotein, fat oxidation, oral fat tolerance test

## Abstract

Ingestion of low glycemic index (LGI) carbohydrate (CHO) before exercise induced less insulin response and higher fat oxidation than that of high GI (HGI) CHO during subsequent exercise. However, the effect on the subsequent postprandial lipid profile is still unclear. Therefore, the aim of this study was to investigate ingestion of CHO drinks with different GI using fructose and glucose before endurance exercise on the subsequent postprandial lipid profile. Eight healthy active males completed two experimental trials in randomized double-blind cross-over design. All participants ingested 500 mL CHO (75 g) solution either fructose (F) or glucose (G) before running on the treadmill at 60% VO_2_max for 1 h. Participants were asked to take an oral fat tolerance test (OFTT) immediately after the exercise. Blood samples were obtained for plasma and serum analysis. The F trial was significantly lower than the G trial in TG total area under the curve (AUC; 9.97 ± 3.64 vs. 10.91 ± 3.56 mmol × 6 h/L; *p* = 0.033) and incremental AUC (6.57 ± 2.46 vs. 7.14 ± 2.64 mmol/L × 6 h, *p* = 0.004). The current data suggested that a pre-exercise fructose drink showed a lower postprandial lipemia than a glucose drink after the subsequent high-fat meal.

## 1. Introduction

An increase in postprandial plasma triacylglycerol (TG) concentrations was suggested to cause damage on vascular subcutaneous cells and vascular walls [1]. The postprandial lipemia phenomena may last for 6 to 8 h [2], suggesting that a high postprandial TG concentration in the circulation is likely lasting an entire day after breakfast is ingested. A number of studies indicated that an increase in postprandial TG level correlates positively with the mortality rate and risk of cardiovascular disease (CVD) [3,4,5]. In order to reduce high postprandial TG concentrations, several studies proposed that endurance exercise was effective for lowering postprandial TG concentrations [6,7,8]. 

A high-carbohydrate (CHO) diet increases the storage and use of muscle glycogen, and improves exercise performance [9,10]. However, a high CHO diet might cause a rise in very-low-density lipoprotein (VLDL) concentration [11,12,13] and a reduction in the level of high-density lipoprotein cholesterol (HDL-C) [14], which were considered to increase the risk of CVD. Previous study showed exercise intervention might elevate the level of HDL-C, increase lipoprotein lipase (LPL) activity [15], increase the transportation of blood lipids into the muscle cells for storage or use, and lower postprandial TG concentrations [11,16]. Katsanos and colleagues [17] showed that following moderate-intensity endurance exercise there was significantly lower insulin concentration and TG area under the curve (AUC) over 6 h after ingestion of a high-fat meal when compared to the no exercise trial. Therefore, insulin action may play one of the key factors in influencing postprandial TG levels [18,19]. 

A rise in insulin concentration by following CHO ingestion results in an increase in the rate of CHO oxidation, and the rate of fat oxidation inhibited [18]. Previous studies examined ingesting CHO meals with a low glycemic index (LGI) and a high glycemic index (HGI) before exercise on substrate utilization during exercise [20,21,22]. The results suggested that ingestion of the LGI meal induced lower insulin response and showed a significantly higher rate of fat oxidation than that of the HGI meal during subsequent exercise [20,22]. Ingesting CHO with a distinct GI stimulates insulin response, leading to changes in the fat oxidation rate during exercise, which possibly exerts varying degrees of influence on postprandial lipid metabolism when the body is recovering from the exercise. Kaviani and colleagues [23] reported that ingestion of a post-exercise LGI meal induced lower postprandial TG concentrations when compared to that of the HGI meal. However, how the pre-exercise CHO influenced the subsequent postprandial lipid profile is still unclear.

An extant study verified that exercise intervention effectively lowered the increased level of blood lipids due to CHO ingestion [24,25]. We hypothesize that ingestion of an LGI drink prior the exercise may induce higher fat oxidation than ingestion of an HGI drink during exercise and subsequently induces a higher plasma TG clearance rate during the postprandial period after a high fat meal. To date, no studies have been conducted on postprandial lipid profiles immediately after exercise with ingestion of pre-exercise GI CHO. Therefore, the purpose of the present study was to determine the effect of fructose versus glucose pre-exercise drinks and exercise intervention on the subsequent postprandial lipid profile.

## 2. Materials and Methods

Eight healthy active males voluntary participated in the present study (age 23.1 ± 0.7 years, weight 68.9 ± 2.0 kg, and maximal oxygen consumption (VO_2_max) 47.7 ± 1.6 mL/kg/min). This study was conducted in the Sports Science Research Center of National Taiwan University of Sport, with the approval of the Human Subject Committee of National Taiwan University of Sport (NTCPE-95-01). Participants were given their written informed consent after complete understood the study design and possible risks. All participants completed the health history questionnaire before undertaking the experiments. 

### 2.1. Experimental Design

A randomized double-blind cross-over experimental design was adopted and the trial order was counter-balanced for this study. All of the participants underwent two experimental trials separated at least seven days. The participants were asked to ingest CHO-containing either a fructose or glucose drink 30 min before running on the treadmill for 1 h at 60% VO_2_max. Immediately after the exercise the participants were asked to take an oral fat tolerance test (OFTT), which asked participants to ingest a high-fat meal to observe postprandial lipemia for 6 h [26]. The participants were asked to record their diet for three days before the first trial and were required to repeat the same diet before the next trial. A standardized lunch and dinner was served for the participants on the day before the main trial. The participants were asked to avoid any heavy physical activity or exercise three days before the main trials. In addition, they were asked to refrain from smoking and ingesting alcohol- and caffeine-containing beverages before the experiment.

### 2.2. Preliminary Measurements

Two preliminary tests were conducted: running economy (RE) test and VO_2_max measurement.

RE test: The RE protocol was a four-stage test running on a treadmill (Medtrack ST65, Quinton, Seattle, Washington, USA) at an initial speed of 7.0–8.0 km/h and increase at 1.0–1.5 km/h every 4 min. The oxygen uptake (VO_2_) was measured 1 min before the end of each stage using a gas analyzer (Vmax Series 29C, Sensor Medics, Yorba Linda, CA, USA). The four VO_2_ measurements were substituted in a linear regression equation to calculate the relationship between VO_2_ and running speed [22]. 

VO_2_max test: The speed of the treadmill was set at a constant pace. The initial slope of the treadmill was set at 3.5% and increased at 2.5% every 3 min. Participants were encouraged to complete every stage of the exercise until volitional fatigue. The VO_2_max criteria were a plateau in VO_2_, heart rate coming close to the age-predicted maximal heart rate, and respiratory exchange ratio (RER) ≥ 1.15.

### 2.3. Test Drink, Oral Fat Tolerance Test, and Lunch and Dinner before Experiment Day

Participants ingested different GI carbohydrate drinks that we provided in the present study: (a) a high-GI drink (GI = 100; HGI); and (b) a low-GI drink (GI = 40; LGI). Carbohydrate source: HGI was 75 g glucose (Wako Pure Chemical Industries, Ltd., Osaka, Japan), or LGI was 75 g fructose (Shimakyu’s Pure Chemicals, Osaka, Japan) in 500 mL water.

An oral fat tolerance test meal included white bread, whipping cream, nuts, butter, and cereal. The meal provided as based on the body weight of the participants and contained 1.2 g/kg fat, 1.1 g/kg CHO, 0.33 g/kg protein, and 16.5 kcal/kg [26]. All of the foods were purchased from the same supermarket, and the foods were of the same brand. The calorie value of the foods, as well as CHO, protein, and fat contents, were calculated according to the nutritional label by the manufacturer. 

The standardized lunch and dinner before the main trial were described in the previous study [26] and were purchased from the same convenience store. The lunch provided 840.0 ± 57.0 kcal, with 50.7 ± 0.3% energy from carbohydrate (106.5 ± 7.4 g), 31.5 ± 0.5% from fat (29.4 ± 1.8 g), and 17.8 ± 0.5% from protein (37.5 ± 3.2 g). The standard dinner offered 692 kcal, with 50% energy from carbohydrate (86.5 g), 32% from fat (24.6 g), and 18% from protein (31.1 g). The calculated GI value was 68.8.

### 2.4. Protocol

Participants were given the same lunch and dinner one day before the start of the experiment. They were instructed to report to the laboratory after overnight fasting for 12 h. After their height and weight were measured, they were asked to ingest a fructose (F) or glucose (G) drink (500 mL). Following 30 min of a resting period, the participants ran at 60% VO_2_max for 1 h on the treadmill. After the exercise was completed, fasting blood specimens were drawn at 0 h from the antecubital vein by a catheter. Subsequently, the participants were asked to ingest an OFTT meal within 20 min, and their blood specimens were collected at 0.5, 1, 2, 3, 4, 5, and 6 h after the meal. The participants were required to sit quietly in the laboratory to avoid any physical activities during the 6 h postprandial period. The environmental temperature was maintained at 22 °C–25 °C and a humidity of 50–60%.

### 2.5. Blood Sample Collection and Analysis

A catheter (Venflon 20G, Ohmeda, Sweden) was connected to the three-way stopcock (Connecta Ltd., Helsingborg, Sweden) with a 10-cm long tube for collecting 10 mL blood samples at each time point. The 10-cm long blood tube was regularly washed with sterile sodium chloride solution (0.9% *w*/*v*) to prevent blood coagulation in the tube. A non-heparinized tube was used to collect 2 mL of blood sample, and it was allowed to stand for 1 h to wait for the blood to coagulate. Another tube containing ethylenediaminetetraacetic acid (EDTA) was used to collect 8 mL of blood sample. The collected sample was then centrifuged (Eppendorf 5810, Hamburg, Germany) at 4 °C at 2000 rpm for 20 min. The extracted serum and plasma samples were stored at −70 °C in a freezer before analysis. Plasma glucose (Shino, Tokyo, Japan), TG (Wako, Osaka, Japan), non-esterified fatty acid (NEFA; Wako, Neuss, Germany), glycerol (Randox, Co., Antrim, UK), and HDL-C (Kyowa, Osaka, Japan) were measured using an automated biochemical analyzer (Hitachi 7020, Tokyo, Japan). Electrochemiluminescence (Elecsys 2010, Roche Diagnostics, Basel, Switzerland) immunoassay was used to analyze the serum insulin concentrations (Roche Diagnostics, Mannheim, Germany). The intra-assay coefficients of the variation of the blood sample measurement were: TG: CV(%) = 4.9; insulin: CV(%) = 2.8; NEFA: CV(%) = 4.51; glycerol: CV(%) = 6.42; glucose: CV(%) = 6.9; and HDL-C: CV(%) = 4.9. 

### 2.6. Statistical Analyses

All collected data was presented as mean ± SD. Changes in blood samples were analyzed by a two-way ANOVA with repeated measures. The Bonferroni post hoc test for comparison with two groups for each time point was used when the ANOVA showed a significant interaction effect (condition × time). The blood biochemistry concentrations over the time AUCs were analyzed using a paired T-test. The differences between F and G trials were evaluated by Cohen’s effect size (ES). The analysis was performed with SPSS 23.0. A *p*-value less than 0.05 was considered statistically significant.

## 3. Results

### 3.1. Plasma Triacylglycerol

**Figure 1:**
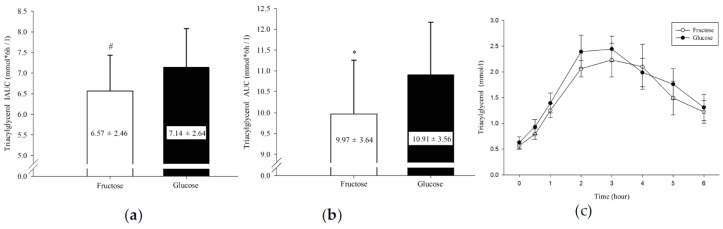
Triacylglycerol (TG) incremental area under the curve (**a**) and TG area under the curve (**b**) in 6 h and postprandial TG concentration over 6 h (**c**). ^#^ F was significantly lower than G (*p* = 0.004). * F was significantly lower than G (*p* = 0.033).

Plasma TG IAUC (Figure 1a) was significantly lower in the F than in the G trial (6.57 ± 2.46 vs. 7.14 ± 2.64 mmol/L × 6 h, *p* = 0.004); plasma TG total AUC (Figure 1b) was significantly lower in the F compared with the G trial (9.97 ± 3.64 vs. 10.91 ± 3.56 mmol/L × 6 h, *p* = 0.033). Plasma TG concentration over 6 h (Figure 1c) showed no significant difference between trials by time interaction (condition × time, *p* = 0.628; condition, *p* = 0.342; time, *p* < 0.001). 

### 3.2. Serum Insulin and Plasma Glucose

**Figure 2:**
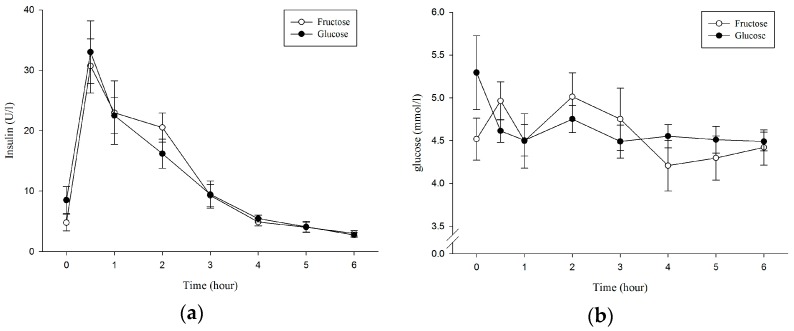
Serum insulin concentrations (**a**) and plasma glucose concentrations (**b**) during the 6 h postprandial period, *p* < 0.05.

Serum insulin concentration and plasma glucose concentration over the 6 h are displayed in Figure 2. There was no significant difference in serum insulin (condition × time, *p* = 0.557; condition, *p* = 0.893; Figure 2a). Plasma glucose concentration showed no significant difference between trials (condition × time, *p* = 0.191; condition, *p* = 0.763; Figure 2b). There were no significant differences between trials in serum insulin AUC and glucose AUC (*p* = 0.717; *p* = 0.951; Table 1).

### 3.3. Plasma Non–Esterified Fatty Acids (NEFA), Glycerol

**Figure 3:**
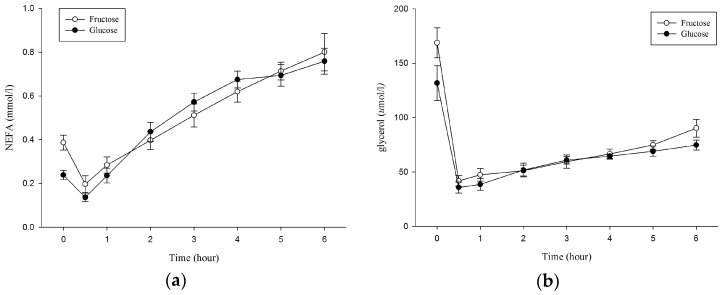
Plasma NEFA concentrations (**a**); and glycerol concentrations (**b**) during the 6 h postprandial period. NEFA: non-esterfied fatty acid.

Plasma NEFA concentration was significantly higher before OFTT (after exercise) in the F trial than in the G trial (0.39 ± 0.1 vs. 0.24 ± 0.06 mmol/L; *p* = 0.011; Table 2). Plasma NEFA concentration over 6 h showed no significant difference between trials (condition × time, *p* = 0.052; condition, *p* = 0.563; time, *p* < 0.001; Figure 3a). There was no difference between trials in plasma NEFA AUC (F: 3.05 ± 0.45; G: 3.06 ± 0.54, *p* = 0.962; Table 1).

Plasma glycerol concentration was significantly higher before OFTT (after exercise) in the F trial than in the G trial (168.75 ± 38.86 vs. 131.75 ± 45.30 μmol/L; *p* = 0.015; Table 2). Plasma glycerol concentration over 6 h showed no significant difference between trials (condition × time, *p* = 0.141; condition, *p* = 0.064; time, *p* < 0.001; Figure 3b). There was no difference between trials in plasma glycerol AUC (F: 395.84 ± 69.55; G: 363.19 ± 64.67, *p* = 0.192; Table 1).

### 3.4. Plasma High-Density Lipoprotein Cholesterol

**Figure 4:**
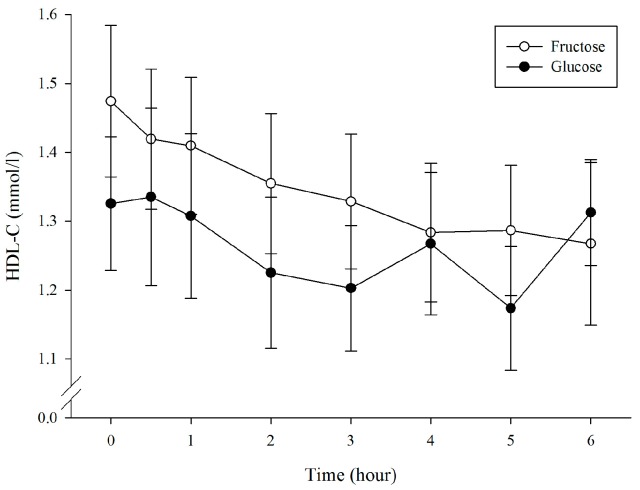
Plasma HDL-C concentrations during the 6 h postprandial period.

Plasma HDL-C concentrations (Figure 4) showed no significant difference between trials by time interaction (condition × time, *p* = 0.336; condition, *p* = 0.118; time, *p* = 0.021). Plasma HDL-C AUC was significantly higher in the F trial compared to the G trial (8.02 ± 1.68 vs. 7.49 ± 1.52 mmol/L × 6 h, *p* = 0.003; Table 1).

## 4. Discussion

The major finding of this study is that ingestion of fructose before endurance exercise lowered subsequent postprandial plasma TG concentrations compared to that of the glucose drink. Several studies demonstrated that endurance exercise effectively reduced postprandial lipemia [7,8,17,25,27]. To our knowledge, no studies have been conducted on how pre-exercise CHO with different GI and endurance exercises influence the subsequent lipid profile after oral ingestion of a high-fat meal.

A previous study reported that while a lower insulin level was induced by ingesting an LGI meal before exercise, a lower CHO oxidation rate was observed compared with when an HGI meal was ingested during exercise [20,22]. After ingestion of the HGI CHO meal, the rise in insulin level, in turn, decreases the fat oxidation rate during exercise, thereby inhibiting exercise-induced lipid metabolism [22,28]. Although we did not measure the RER to examine the rate of substrate utilization during exercise, the plasma NEFA and glycerol concentrations of the F trial was significantly higher than that of the G trial following 60 min of exercise (Figure 3). The current result is similar to previous studies, which might indicate a higher fat oxidation occurred in the F trial during exercise in the current study [20,22]. Therefore, the current study supported those of previous studies that, after ingesting a CHO diet with different GI and engaging in exercise, the LGI trial showed a significantly higher fat oxidation rate during the exercise than did the HGI trial [20,21,22]. This result verifies that ingesting an LGI CHO drink before exercise could depress lipid metabolism less during exercise compared with the HGI CHO drink. The higher fat oxidation occurring in the F trial during exercise might result in a higher plasma TG clearance rate during subsequent postprandial period.

Previous study demonstrated that the additional insulin administration after ingestion of high fat meal showed an improvement in postprandial TG disposal in type I diabetes [29]. The study indicated that the insulin concentration played an important role on postprandial TG removal. However, the current study did not find differences in insulin concentration between trials during OFTT. This might be due to the exercise before OFTT diminishing the difference in postprandial insulin response even though we fed different GI CHO before exercise.

In the present study, the postprandial TG AUC and IAUC in the F trial demonstrated significantly lower values than the G trial (Figure 1). An increase in the plasma TG removal rate is possibly the factor causing a reduction in the plasma TG level, including decreasing the release of liver TG and increasing the transport of TG into muscle cells, or storage and utilization [24,30,31]. Ingestion of fructose was thought to increase postprandial lipemia in a sedentary population. Chong and colleagues [32] concluded that ingestion of fructose induced lower insulin secretion and might result in less activation of lipoprotein lipase (LPL), which consequently leads to impairing TG clearance. However, the negative effect may be offset by increasing physical activities [33]. Interestingly, Egli and colleagues [34] reported exercise prevented short-term high-fructose diet induced hypertriglyceridemia and increased lipid oxidation. After exercise, muscle LPL activity is increased, stimulating the transport of TG into the muscle cell for storage and utilization, which may reduce plasma TG concentration [24,35]. A previous study determined that a single session of exercise significantly enhanced the muscle LPL activity [36,37]. Seip and colleagues [38] also found that the expression of LPL genes in fat tissues did not differ significantly after exercise, which further reflects the importance of muscle to blood lipid metabolism after exercise. However, the different insulin responses caused by ingestion of CHO solution with different GI were likely to influence the muscle LPL activity [36,39]. In addition, the levels of glycogen and insulin increased considerably following ingestion of CHO; however, LPL activity was significantly decreased [40,41]. Seip and colleagues [42] demonstrated that the LPL mRNA level was significantly increased after 4 h of exercise, while lower insulin level was observed. This result caused a rise in VLDL concentration and a reduction in HDL-C release [39,43]. Another study reported that glucose ingestion elicits an insulin response that is evidently more apparent than that of fructose ingestion [44,45]. Moreover, the fat oxidation rate during exercise is relatively higher after fructose ingestion [46], which also influences LPL activity, eliciting changes in the postprandial TG level. This probably partially explains why the F trial postprandial TG level was significantly lower than that of the G trial in the current study.

The postprandial HDL-C level is related to the metabolic rate of TG-rich lipoprotein [24], and exercise may promote a rise in HDL-C concentration [47,48]. However, HDL-C concentration could be influenced by the insulin level [49]. The result of the present study indicated that when the participants ingested the F drink before exercise, the significantly higher postprandial HDL-C level was observed compared with when ingesting the G solution (Figure 4). A previous study reported when CHO ingestion was controlled for four weeks, the fasting insulin level increased significantly, and the HDL-C level was significantly lower than the pretest value [50]. Another study compared ingestion of different concentrations of CHO beverages and found that low CHO intervention resulted in higher HDL-C concentration, which was effective for triggering a decrease in the postprandial TG level [51]. Compared with ingesting the F drink, ingesting the G drink induced higher insulin response which, in turn, caused a reduction in the postprandial HDL-C level, thereby weakening the TG removal capability.

Previous studies have largely explored the relationship between exercise intervention and postprandial lipid metabolism. No studies have investigated ingestion of different GI CHO before exercise generating an influence on the postprandial lipemia immediately after exercise. This is the first study to elucidate the interactive effects of different GI drinks and exercise intervention on lipid metabolism following OFTT. We found that intervention might exert a retention effect, suggesting that ingesting a CHO drink with different GI before exercise influences the rate of substrate utilization during exercise, as well as the postprandial lipid level when OFTT is ingested after exercise.

### Limitations

One of the major limitation of the present study was that the participants were given 75 g glucose or fructose drinks which were not adjusted by their body weight, although the different body size may have resulted in different magnitudes of glycemia when ingesting of the same amount of CHO. However, the design of the present study was mainly to induce different glycemic and insulinemic responses via different GI carbohydrate prior to exercise in order to influence the subsequent substrate utilization. In addition, the present study design was a with-in subject design. Therefore, we speculate that even if we adjust the amount of pre-exercise CHO by the subject’s body weight, the outcome will possibly be similar to the present results.

## 5. Conclusions

This study found that when fructose was ingested before endurance exercise, the fructose trial significantly lowered TG AUC and IAUC compared with the glucose trial after OFTT. This result is possibly related to the pre-exercise low insulin level that induced higher fat oxidation during exercise. However, the mechanism involved remains elusive and warrants further investigation in the future.

## Figures and Tables

**Table 1:** The plasma and serum sample concentrations area under the curve.

	Fructose	Glucose	*p*	ES
Insulin (μU/mL × 6 h)	74.06 ± 20.95	71.73 ± 17.88	0.717	0.12
TG (mmol/L × 6 h)	9.97 ± 3.64	10.91 ± 3.56	0.033 *	0.26
TG IAUC(mmol/L × 6 h)	6.57 ± 2.46	7.14 ± 2.64	0.004 *	0.22
Glucose (mmol/L × 6 h)	27.46 ± 3.30	27.56 ± 1.59	0.951	0.04
NEFA (mmol/L × 6 h)	3.05 ± 0.45	3.06 ± 0.54	0.962	0.02
Glycerol (μmol/L × 6 h)	395.84 ± 69.55	363.19 ± 64.67	0.192	0.49
HDL-C (mmol/L × 6 h)	8.02 ± 1.68	7.49 ± 1.52	0.003 *	0.33

* Significant difference between F and G (*p* < 0.05). Values are mean ± SD. TG: triacylglycerol; IAUC: incremental area under the curve; NEFA: non–esterified fatty acids; HDL-C: high density lipoprotein cholesterol.

**Table 2:** The plasma and serum sample concentrations before OFTT.

	Fructose	Glucose	*p*	ES
Insulin (μU/mL)	4.78 ± 3.86	8.50 ± 6.31	0.177	0.71
TG (mmol/L)	0.57 ± 0.21	0.63 ± 0.31	0.580	0.23
Glucose (mmol/L)	4.52 ± 0.69	5.29 ± 1.22	0.101	0.78
NEFA (mmol/L)	0.39 ± 0.10	0.24 ± 0.06	0.011 *	1.82
Glycerol (μmol/L)	168.8 ± 38.86	131.7 ± 45.30	0.015 *	0.88
HDL-C (mmol/L)	1.47 ± 0.31	1.33 ± 0.27	0.184	0.48

* Significant difference between F and G (*p* < 0.05). Values are mean ± SD. TG: triacylglycerol; NEFA: non–esterified fatty acids; HDL-C: high density lipoprotein cholesterol.
